# Inhibition of nitric oxide production reverses diabetes-induced Kupffer cell activation and *Klebsiella pneumonia* liver translocation

**DOI:** 10.1371/journal.pone.0177269

**Published:** 2017-05-11

**Authors:** Shu-Han Lin, Pei-Hsuan Chung, Ying-Ying Wu, Chang-Phone Fung, Ching-Mei Hsu, Lee-Wei Chen

**Affiliations:** 1 Institute of Emergency and Critical Care Medicine, National Yang-Ming University, Taipei, Taiwan; 2 Department of Nursing, National Taiwan University Hospital, Taipei, Taiwan; 3 Department of Biological Sciences, National Sun Yat-Sen University, Kaohsiung, Taiwan; 4 Department of Surgery, Kaohsiung Veterans General Hospital, Kaohsiung, Taiwan; 5 Division of Infectious Diseases, Department of Medicine, Taipei Veterans General Hospital, Taipei, Taiwan; Universidad Francisco de Vitoria, SPAIN

## Abstract

*Klebsiella pneumoniae* (KP) is the most common pathogen of pyogenic liver abscess in East and Southeast Asia and diabetes mellitus (DM) is a major risk factor. The effect and mechanism of diabetes on KP liver abscess was examined in streptozotocin-induced diabetic mice and Akita mice (C57BL/6J-*Ins*2^Akita^). KP translocation to liver and plasma alaine transaminase levels were increased and liver clearance of KP was decreased in DM mice. Diabetic mice exhibited overgrowth of *Enterococcus* as well as *E*.*coli* and decreased *lactobacilli/bifidas* growth in intestine, increased intestinal iNOS protein and nitrite levels in portal vein, and increased IL-1β and TNF-α expression of Kupffer cells. Fructooligosaccharides (FOS) or dead *L*. *salivarius* (dLac) supplementation reversed diabetes-induced enteric dysbiosis, NO levels in portal vein, and KP translocation to liver. L-NAME treatment decreased intestinal iNOS protein expression as well as Kupffer cell activation and increased liver clearance of KP in DM mice. Dead *E*.*coli* (2×10^8^ CFU/ml) feeding for one week induced iNOS and TLR4 expression of intestine in germ-free (GF) mice. Dead bacteria feeding induced IL-1β and TNF-α expression of Kupffer cells in GF mice but not in GF TLR4^-/-^ mice. In conclusion, balance of intestinal microflora is important for preventing intestinal iNOS expression, Kupffer cell activation, and KP liver translocation in diabetes. Reversal of diabetes-induced enteric dysbiosis with FOS or dead *L*. *salivarius* decreases diabetes-induced intestinal iNOS expression and KP liver translocation. Diabetes induces Kupffer cell activation and KP liver translocation through enteric dysbiosis and nitric oxide production.

## Introduction

*K*. *pneumoniae* is a Gram-negative, non-motile, encapsulated, lactose fermenting, facultative anaerobic, rod shaped bacterium found in the normal flora of the mouth, skin, and intestine [1]. In western countries, pyogenic liver abscess is usually a polymicrobial infection caused by *Escherichia coli* (*E*. *coli*), streptococci, and anaerobic bacteria. However, over the past two decades in East and Southern Asia, *K*. *pneumoniae* is the main etiological agent [2]. Moreover, DM is the most common underlying condition, with a prevalence ranging from 45% to 75% in patients with *Klebsiella pneumoniae* liver abscess [1]. Previous studies in Taiwan, Singapore and Korea found a high prevalence of the capsular polysaccharides (CPS) K antigen serotypes K1 (54.5–63.4%) and K2 (5–21.2%) of *K*. *pneumoniae* in pyogenic liver abscess isolates [3]. However, there is limited knowledge regarding the pathological mechanisms of how this bacterium infects the liver in diabetic patients.

Alterations of intestinal microbiota seem to play an important role in induction and progression of liver damage [4]. Gut flora alterations consist of overgrowth and release in the circulation of bacterial endotoxins (e.g., bacterial lipopolysaccharide (LPS), peptidoglycan, lipoproteins, and various lipopeptides). TLRs, acting as pathogen sensors, contribute to adaptive immune response and regulation of inflammation and represent a link between intestinal flora changes, endotoxemia, and liver damage [5]. Several studies, in experiment alcohol-induced liver disease, postulated that LPS binds to hepatic Kupffer cells via TLR4 with resulting induction of TNF-α to induce hepatocyte damage [6]. Previously, we have demonstrated a significant 3.5-fold increase of plasma endotoxin levels of the portal vein in diabetes mice [7]. Different mouse models revealed that inflammasome-deficiency-associated changes in the configuration of the gut microbiota are associated with exacerbated hepatic steatosis and inflammation through influx of TLR4 and TLR9 agonists into the portal circulation, leading to enhanced hepatic tumor-necrosis factor (TNF-α) expression that develop chronic hepatic inflammation, non-alcoholic steatohepatitis (NASH) [8]. A recent study of NASH showed that the production of IL-1β by Kupffer cells induced by TLR9 signaling results in hepatic steatosis, inflammation, and fibrosis [9]. However, the involvement of IL-1β and TNF-α of Kupffer cells in diabetes-enhanced liver abscess has not been clarified.

Prebiotics are non-digestible short-chain oligosaccharides which enter colon and are fermented to change the GI environment (acid pH and increased shot-chain fatty acid) to selectively stimulate the growth of certain commensal bacteria such as *bifidocateria* and *lactobacillus* [10]. Effects of prebiotic and probiotic supplementation on *K*. *pneumoniae*-induced liver abscess have not been well studied.

At normal levels, nitric oxide (NO) is a key mediator of intestinal cell and barrier function [11]. When NO is present in excess, however, the result is barrier dysfunction [12]. Another recent study reported that iNOS inhibitors can protect the liver against the injurious effects of chronic alcohol and iNOS may be a useful target for prevention of alcoholic liver disease [13]. Intestinal dysbiosis and BT are common in patients with advanced liver disease, and there are strong evidences that the translocation of bacteria and their products across the epithelial barrier drives experimental liver disease progression [14]. Furthermore, intestinal NO has been shown to function as an inducer for the interorgan immune communication between gut and liver, but it remains unknown whether intestinal NO also directly manipulates hepatic innate immune system to trigger release of cytokines by Kupffer cells which result in trapping of bacteria in liver. Thus, the aims of this study were to examine the mechanisms of diabetes-induced *Klebsiella pneumoniae* liver abscess. Also, the correlation between the changes of intestinal microbiome and progression of liver injury as well as inflammatory cytokines of the Kupffer cells in diabetic mice were assessed. Finally, possible therapeutic interventions to decrease Kupffer cells activation and *Klebsiella pneumoniae* liver translocation in diabetic mice were evaluated.

## Materials and methods

### Animals and treatments

Specific pathogen-free (SPF) (total n = 360) and germ-free C57BL/6J (wild-type, WT) mice (total n = 90) weighing between 18 g and 25 g were purchased from the National Laboratory Breeding and Research Center (NLBRC, Taipei, Taiwan). Ins2-Akita (Ins2^Akita^ mutation mutant) mice (C57BL/6J background) (total n = 120) were purchased from the Jackson Laboratory (Bar Harbor, ME). The Ins2^Akita^ mutation results in a single amino acid substitution in the insulin 2 gene that causes misfolding of the insulin protein[15]. Male mice heterozygous for this mutation have progressive loss of β-cell function and significant hyperglycemia, as early as 4 weeks of age. To develop a diabetic mouse model, male C57BL/6 mice were given one intraperitoneal (i.p.) injection of streptozotocin (STZ, Sigma-Aldrich) to induce the death of pancreatic β cells. STZ was freshly dissolved in dilution buffer (0.1 M sodium citrate, pH 4.5, titrated with HCl and stored at 4°C) and sterilized. To induce diabetes, mice were fasted for 24 hr before STZ injection and STZ was given (150 mg/kg of body weight). Mice with two consecutive readings of blood glucose >250 mg/dl were considered diabetic. All mice had ad libitum access to food and water and were fed a standard laboratory diet (1324 TPF; Atromin; Large Germany; 11.9 kJ/g, 19% crude protein, 4% crude fat, 6% crude fiber).

To investigate the association of intestinal dysbiosis with the increased BT to liver (especially the translocation of *K*. *pneumoniae*), prebiotic fructooligosaccharides (FOS, Sigma) were given in drinking water to mice (250 mg in 100 ml) to stimulate the growth of probiotic bacteria for one week. To confirm that the mechanisms for improvement and prevention by FOS supplementation are through increasing specific groups of intestinal commensal microbiota (*Lactobacillus* or *Bifidobacteria*) to reduce bacterial translocation (especially *K*. *pneumonia*) to liver, diabetic mice were fed with dead *Lactobacillus salivarius* CECT5713 [16] (2×10^8^ CFU/ml) in drinking water for one week. *Lactobacillus salivarius* were killed by heating at 63°C for 30 minutes. The water with FOS or bacteria was refreshed every day. To determine whether there is a potential link between intestinal nitric oxide production and hepatic bacterial clearance, STZ-DM mice were fed with an L-NAME (L-NG-Nitroarginine Methyl Ester, 0.5 mg/ml) or D-NAME (0.5 mg/ml, as a control drug) in drinking water for one week. L-NAME is a NO synthase inhibitor and inhibits the production of NO by inducible NO synthase and constitutive NO synthase [13]. To examine whether bacteria could induce IL-1β expression of liver through toll-like receptors of the intestinal mucosa, germ-free WT mice and germ-free TLR4^-/-^ mice were orally fed with dead *E*.*coli* or *S*. *aureus* (2×10^8^ CFU/ml) for one week.

### Ethics statement

This study was approved by the Institutional Animal Care and Use Committee of Kaohsiung Veterans General Hospital (Permit Number: VGHKS-103-A007), and animal experiments were performed according to Animal Experimentation Regulations of Kaohsiung Veterans General Hospital. All efforts were made to minimize suffering. Animals were checked every 6 hours for signs of distress and endpoints. Specific criteria used to determine when the animals should be euthanized were in accordance with Remick lab report [17]. Mice were systematically euthanized with avertin (15 mg/kg, Sigma) when they were found in a moribund state as identified by inability to maintain upright associated or not with labored breathing and cyanosis.

### Translocation of orally fed K. pneumonia to liver and MLNs

One month after induction, mice were fed with pathogenic *K*. *pneumoniae* (2 × 10^8^ CFU/ml) and GFP-expressing *K*. *pneumoniae* (2 × 10^8^ CFU/ml) respectively for 2 weeks. Liver and MLNs were collected, weighed and homogenized in 1 ml of sterile saline. Aliquots of the homogenates from each sample were plated onto Chrom Orientation plates for growth of pathogenic *K*. *pneumoniae* and onto TSB agar plates for growth of GFP-expressing *K*. *pneumoniae*. The plates were examined for CFU after aerobic incubation at 37°C for 24 hr.

### Bacterial contents of intestinal lumen

The collected contents from the terminal ileum lumen were homogenized in equal volume of sterile saline. The total aerobic bacteria were cultured onto tryptic soy broth (TSB) agar plates (DIFCO); the aerobic bacteria *Enterobacteriaceae* and *Enterococcus* were cultured on EMB and m-Enterococcus agar plates. The anaerobic bacteria *Bacteroides*, *Clostridium perfringens* and *Lactobacillus/Bifidobacterium* were cultured on BBE, TSC and BIM-25 agar plate respectively. The plates were examined for colony forming units (CFU) after aerobic incubation at 37°C for 24 hr and anaerobic incubation at 37°C for 5 days in an anaerobic chamber.

### 16S rRNA gene sequencing and analysis

We extracted genomic DNA of guts from different groups in triplicate and amplified a portion of the V2 region of the 16S rRNA gene of bacteria using barcoded primers, followed by high-throughput sequencing of amplicons. We generated approximately 20,000 high quality sequences per sample. Sequences were demultiplexed and analyzed using the QIIME (Quantitative Insights Into Microbial Ecology) software package [18].

### Bacterial DNA extraction and quantitative Real-Time PCR

Bacterial genomic DNA was extracted from terminal ileum using the Qiagen DNA stool kit according to the manufacturer’s directions. The number of specific bacterial groups was determined by using StepOnePlus^™^ Real-Time PCR System (Applied Biosystems 7300) [19].

### Plasma Alanine Aminotransferase (ALT) assay

A 0.5 ml of blood sample was collected from the portal vein. Plasma samples were assayed for ALT levels using a commercially available analytical kit (Transaminase CII-Test; Wako Pure Chemical Industries).

### Hepatic bacteria clearance of intravenously injected K. pneumoniae

We examined hepatic *K*. *pneumoniae* defense mechanism by injecting *K*. *pneumoniae* to the superior mesenteric vein. 100 μl of normal saline (pH 7.2) containing pathogenic *K*. *pneumoniae* (1 × 10^3^ CFU, K2 serotype) were injected into the branch of superior mesenteric vein (SVC). The liver was collected, weighed and homogenized in 1 ml of sterile saline at 4 hr after the injection. 100 μl of blood were taken from heart. Blood or aliquots of the homogenates were plated onto tryptic soy broth (TSB) agar plates (DIFCO) [20]. The plates were examined after aerobic incubation at 37°C for 24 h.

### Bacterial translocation to liver

The collected liver were weighed and homogenized in equal volume of sterile saline. Aliquots of the homogenates were plated onto TSB agar plates.

### Translocation of intraluminally injected K. pneumoniae

After anesthetizing the animals with avertin (15 mg/kg), the two ends of a 10-cm segment of the small intestine were clipped. 500 μl of normal saline (pH 7.2) containing *K*. *pneumoniae* (5 × 10^7^ CFU) was injected into the isolated intestinal segment. After 2 h, liver was collected, weighed and homogenized in 1 ml sterile saline, and 100 μl of blood were withdrawn from the heart. Blood or aliquots of the homogenates were plated onto TSB agar plates with or without ampicillin (100 μg/ml). The plates were examined after aerobic incubation at 37°C for 24 h. *K*. *pneumoniae* CG43 (a clinical isolate of K2 serotype) and green fluorescent protein (GFP)-expressing *K*. *pneumoniae* are used in this study. *K*. *pneumoniae* CG43 isolated from a patient with pyogenic liver abscess from Taipei Veteran General Hospital in Taiwan. GFP-expressing *K*. *pneumoniae* generated by transformation of pGFP*uv*-Tc (Clonthech) is a gift from Taipei Veteran General Hospital in Taiwan.

### Kupffer cell purification

The liver was perfused *in situ* through the portal vein with Ca^2+^- and Mg^2+^-free phosphate-buffered saline containing 10 mM ethylenediaminetetraacetic acid at 37°C for 5 minutes. Subsequently perfusion was performed with HBSS containing 0.1% collagenase IV (Sigma) at 37°C for 5 minutes. After digestion, the liver was excised and the suspension was filtered. The filtrate was centrifuged twice at 50*g* at 4°C for 1 minute. The supernatant was collected and centrifuged at 300g for 5minutes, and the pellet was resuspended with buffer. The cell suspension was then layered on top of a density cushion of 30%/60% discontinuous Percoll (Pharmacia) and centrifuged at 900g for 15 minutes to obtain the Kupffer cell fraction, followed by washing with the buffer again [21].

### Expression of TNF-α, IL-6, and IL-1β in Kupffer cells

The total RNAs were extracted from Kupffer cells using the Miniprep Purification Kit (GeneMark). Real-time polymerase chain reaction was performed with the SYBR Green PCR Master Mix and ABI PRISM 7700 Sequence Detection Systems (Applied Biosystems, Foster City, CA) according to the manufactu rer’s suggested protocol. Sets of TNF-α, IL-6, and IL-1β primers were designed according to those genes documented in GenBank [22].

### Western immunoblots

The iNOS protein expression in the intestinal mucosa were identified by mouse monoclonal antibodies (R&D Systems) and TLR4 were identified by mouse monoclonal, rabbit polyclonal and goat polyclonal antibodies, respectively (Santa Cruz Biotechnology Inc.).

### Griess reagent assay

A 100 μl of serum was mixed with 40 μl of Griess reagent in each well of the ELISA plate. The mixture was incubated at room temperature for 20 min in the dark and measured for the absorbance at 550 nm. The concentration of NO in serum was determined as compared to the standard curve.

### Statistical analysis

All immunoblotting and electrophoretic mobility shift assays were analyzed by densitometric scanning. All data are analyzed by one-way analysis of variance (ANOVA), followed by Tukey’s Multiple Comparison Test. All values in the figures and text were expressed as mean ± standard error of the mean, and P values of less than 0.05 are considered to be statistically significant.

## Results

### Diabetes induced orally fed K. pneumoniae translocation to liver and MLNs in STZ-DM mice

**Fig 1:**
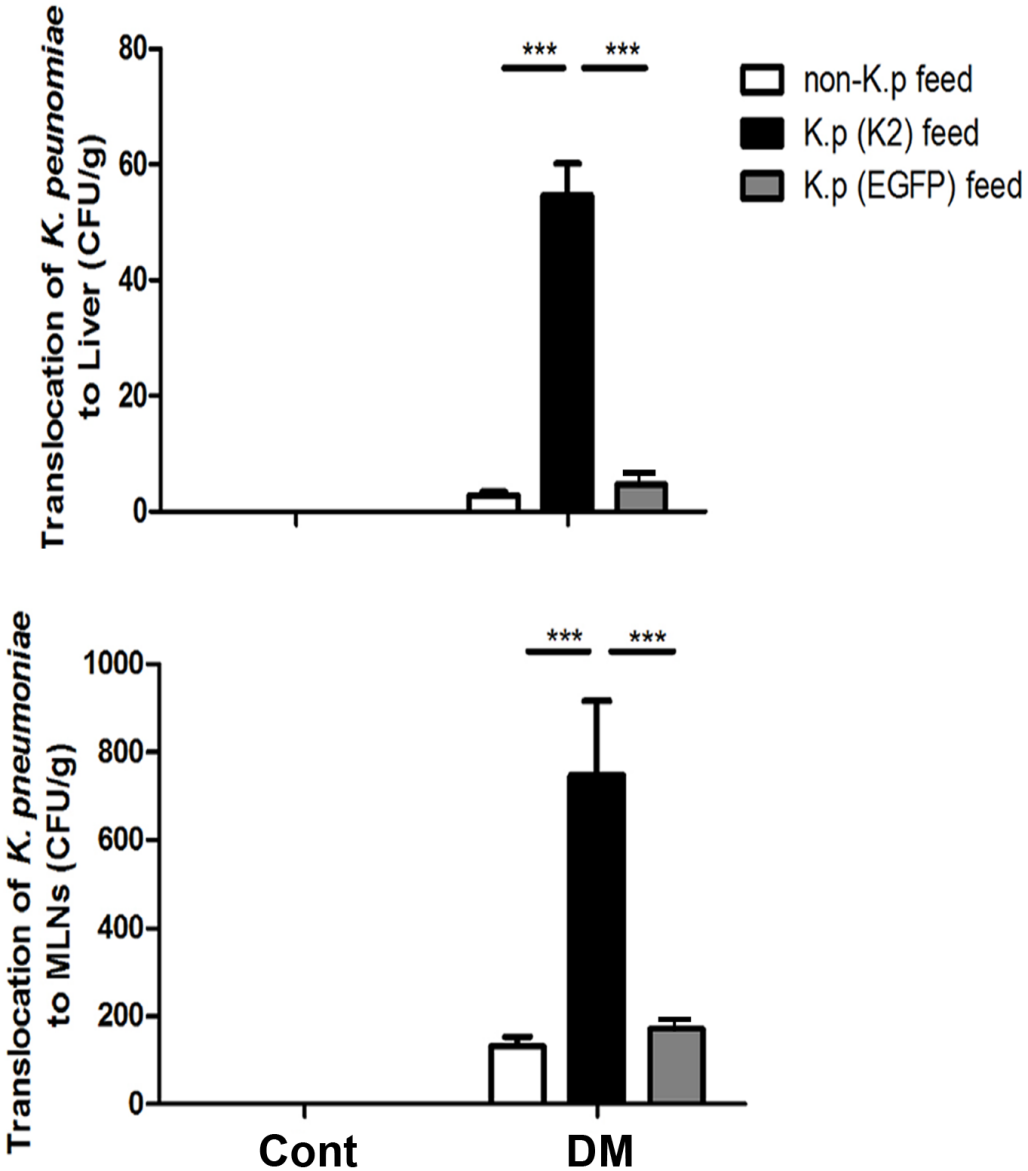
Increased *K*. *pneumoniae* translocation to liver and MLNs in STZ-DM mice. Mice were orally fed with nonpathogenic, pathogenic, or GFP-expressing *K*. *pneumoniae* for 2 weeks. The translocation of *K*. *pneumoniae* to liver and MLNs was examined. Non-pathogenic as well as *K*. *pneumoniae* translocation to liver and MLNs did not happen in the control mice. Translocation of pathogenic *K*. *pneumoniae* (K2) to liver and MLNs was significantly increased in STZ-DM mice as compared with the control group. Translocation of *K*. *pneumoniae* to liver was significantly increased by feeding pathogenic *K*. *pneumoniae* (K2) as compared with non-*K*. *pneumoniae* feeding and GFP-expressing *K*. *pneumonia*e feeding in STZ-DM mice. STZ, streptozotocin; DM, diabetes mellitus; GFP, green fluorescence protein; MLNs, mesenteric lymph nodes. *P<0.05, **P<0.01, ***P<0.001. n = 5/group.

To investigate effect of diabetes on the intestinal *K*. *pneumoniae* translocation to liver and MLNs, STZ-DM mice were respectively fed with nonpathogenic, pathogenic or GFP-expressing *K*. *pneumoniae* for 2 weeks. Non-pathogenic as well as *K*. *pneumoniae* translocation to liver and MLNs did not happen in the control mice (Fig 1). There were significant 19.2-fold and 5.6-fold increases of *K*. *pneumoniae* CG43 translocation to liver and MLNs, respectively, in STZ-DM mice fed with pathogenic *K*. *pneumoniae* compared to those with non-pathogenic *K*. *pneumoniae* feeding (Fig 1A). Because of non-infecting feature, the results of GFP-expressing *K*. *pneumonie* were consistent with the non-pathogenic *K*. *pneumoniae* feeding. These results indicate that diabetes enhances pathogenic *K*. *pneumoniae* translocation to liver and MLNs.

### Diabetes induced intestinal bacterial growth as well as enteric dysbiosis and FOS or dead L. salivarius feeding reversed them

**Fig 2:**
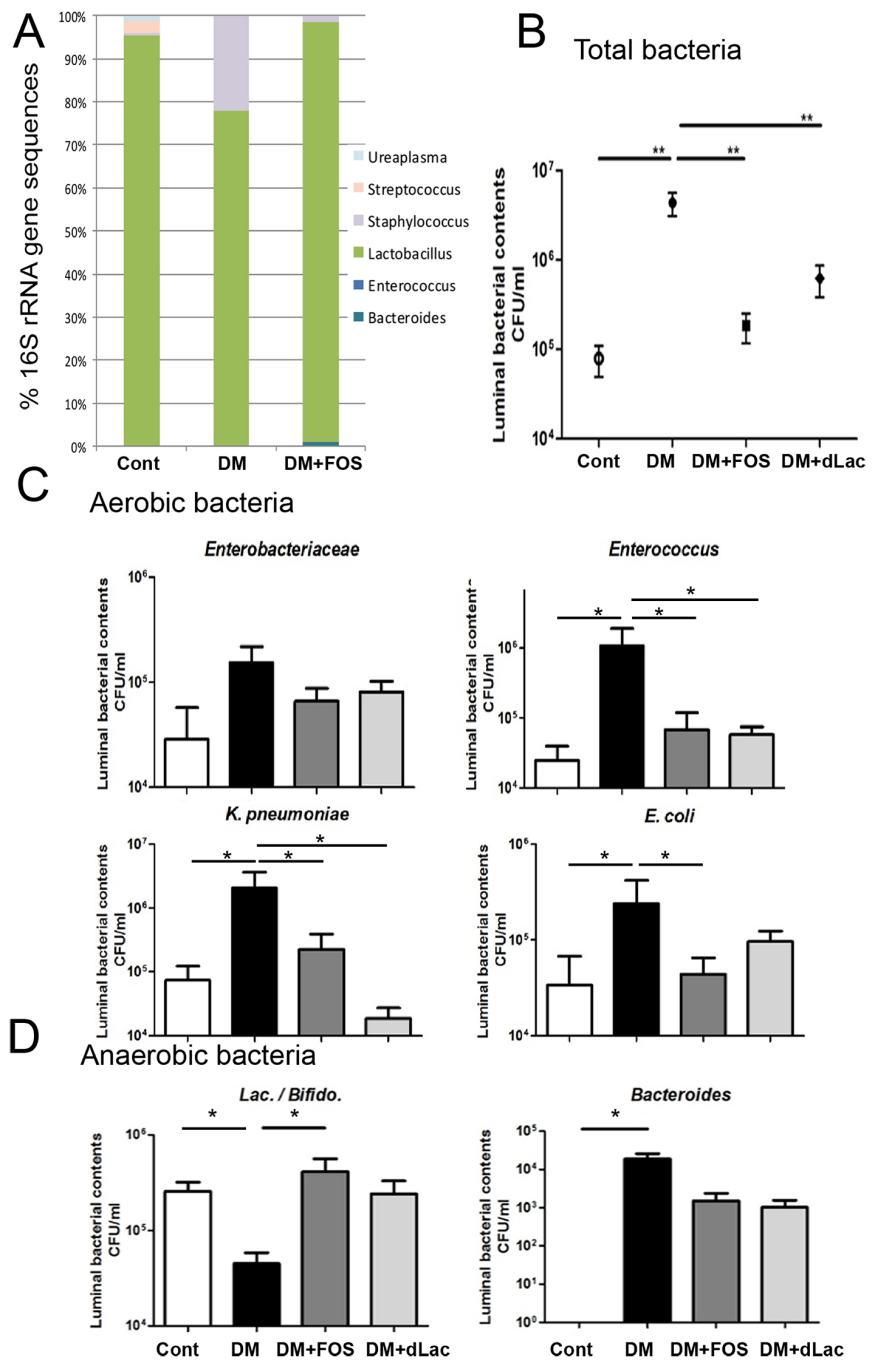
Fructooligosaccharides (FOS) or dead *L*. *salivarius* feeding decreased the intestinal bacterial overgrowth in STZ-DM mice. (A) Relative abundance of bacteria across difference groups, as indicated by 16S rRNA gene sequencing. Values represent the mean abundance of Genus found at >1% relative abundance in at least one sample. (B) The collected mucosa from the terminal ileum were weighed and homogenized. Total bacteria counts were significantly increased in STZ-DM mice and FOS or dead *L*. *salivarius* feeding decreased them. (C) *E*. *coli* and *K*. *pneumoniae* of intestinal lumen were significantly increased in STZ-DM mice in comparison with those in SPF WT mice. FOS or dead *L*. *salivarius* feeding decreased the growth of pathogenic bacteria such as *E*. *coli* and *K*. *pneumoniae* in STZ mice. (D) The growth of *lactobacilli/bifidas* in intestinal lumen was significantly decreased in STZ-DM mice compared with SPF mice. FOS feeding significantly increased them in STZ-DM mice. STZ, streptozotocin; DM, diabetes mellitus; FOS, fructooligosaccharides; dLac, dead *L*. *salivarius*. *P<0.05, **P<0.01. n = 5/group.

To examine the effect of diabetes on enteric microflora, the total bacteria in the lumen of the terminal ileum in STZ-DM was studied. Moreover, to assess whether probiotic or prebiotic treatment could reverse diabetes-induced intestinal bacterial overgrowth and enteric dysbiosis, STZ-DM mice were fed with a prebiotic like FOS or a probiotic like dead *L*. *salivarius* respectively. FOS or dead L. salivarius feeding did not change body weight or blood glucose levels in STZ-DM mice (568±100 mg/dl). High-throughput 16S rRNA gene sequencing revealed that *Lactobacillus* was decreased in DM group as compared with WT group and FOS treatment significantly increased *Lactobacillus* of the intestine in STZ-DM mice (Fig 2A). Diabetes significantly induced the overgrowth of total bacteria in the lumen of small intestine in STZ-DM mice compared with those in control group (Fig 2B). The total aerobic bacterial counts in intestinal lumen had a significant 55-fold increase in STZ-DM mice compared to those in the control group. The counts of aerobic bacteria *Enterobacteriaceae*, *Enterococcus* and *K*. *pneumoniae* in intestinal lumen (Fig 2C) were also increased in STZ-DM mice. The counts of anaerobic *Bacteroides* were significantly increased but the counts of *Lactococcus/Bifidobacterium* were significant decreased in the intestinal lumen (Fig 2D) in STZ-DM mice. There were 96-fold and 86-fold decreases of total bacteria in the lumen in STZ-DM mice by FOS feeding and dead *L*. *salivarius* feeding respectively as compared with those in STZ-DM mice (Fig 2B). In addition, the enteric dysbiosis of aerobic and anaerobic bacteria in the lumen was also reversed by FOS or dead *L*. *salivarius* feeding. Especially, the growth of pathogenic bacteria such as *Enterococcus*, *E*. *coli*, and *K*. *pneumoniae* were decreased in STZ-DM mice by FOS or dead *L*. *salivarius* feeding (Fig 2C). These results suggest that diabetes induces bacteria overgrowth but decrease *Lactococcus/Bifidobacterium* in the lumen of the intestine. FOS or dead *L*. *salivarius* feeding reverses them.

### Diabetes induced liver dysfunction and FOS or dead L. salivarius feeding reversed it

**Fig 3:**
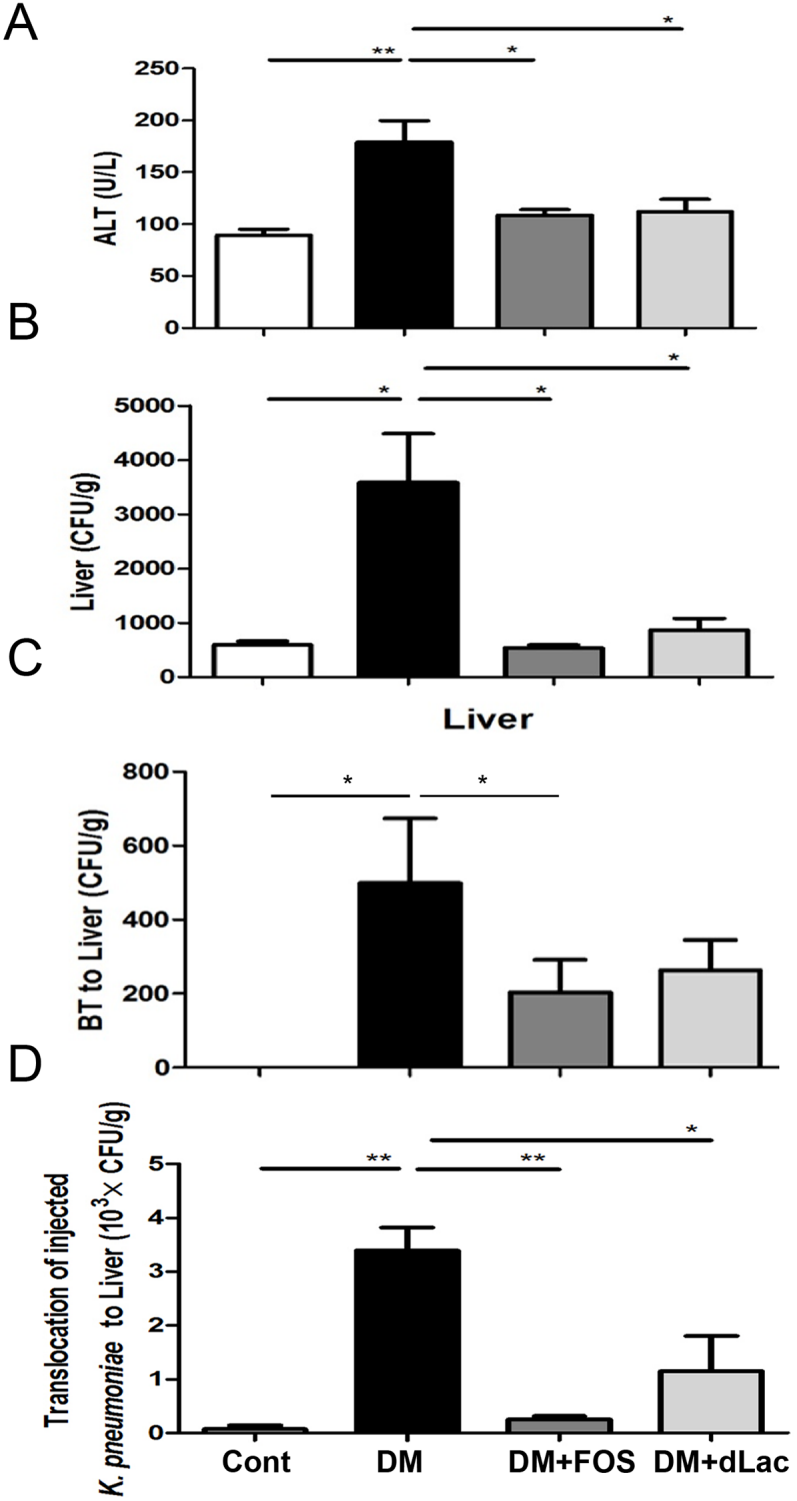
Fructooligosaccharides (FOS) or dead *L*. *salivarius* feeding reduced enteric bacteria as well as *K*. *pneumoniae* translocation to liver and increased hepatic bacteria clearance in STZ-DM mice. (A) The level of serum ALT was significantly increased in STZ-DM mice. FOS and dead *L*. *salivarius* supplementation significantly decreased serum ALT levels in STZ-DM mice. (B) STZ-DM mice demonstrated a significant increase of *K*. *pneumoniae* loads of liver after injection of *K*. *pneumoniae* in the superior mesenteric vein as compared with that in the control group. FOS or dead *L*. *salivarius* feeding significantly decreased the bacterial loads of liver in STZ-DM mice. (C) FOS or dead *L*. *salivarius* feeding decreased diabetes-induced bacterial translocation to liver in STZ-DM mice. (D) FOS or dead *L*. *salivarius* feeding significantly decreased diabetes-induced pathogenic *K*. *pneumoniae* translocation to liver in STZ-DM mice. *K*. *pneumoniae* (5 × 10^7^ CFU in 500 μl of normal saline) was injected into the isolated intestinal segment. After 2 h, liver was collected, weighed, and homogenized in equal volume of sterile saline for culture. ALT, alanine transaminase; BT, bacterial translocation; STZ, streptozotocin; DM, diabetes mellitus; FOS, fructooligosaccharides; dLac, dead *L*. *salivarius*. *P<0.05, **P<0.01, ***P<0.001. n = 6/group.

There was a significant 2.0-fold increase of serum ALT level (Fig 3A) in STZ-DM mice as compared with that in the control group. Moreover, FOS and dead *L*. *salivarius* supplementation significantly decreased serum ALT levels 38% and 39% respectively in STZ-DM mice as compared with those in STZ-DM mice (Fig 3A). These results indicate that diabetes induces liver injury and FOS or dead *L*. *salivarius* feeding reverses it.

### Diabetes induced hepatic bacteria clearance impairment and FOS or dead *L*. *salivarius* feeding reversed it

To directly examine the effect of diabetes on the hepatic defense mechanism, *K*. *pneumoniae* was injected into the branch of superior mesenteric vein (SVC) and hepatic clearance was examined in STZ-DM mice. The bacterial loads of liver showed a significant 5.3-fold increase in STZ-DM mice compared to those in the control mice (Fig 3B), suggesting that the hepatic bacteria clearance was impaired in STZ-DM mice. FOS or dead *L*. *salivarius* supplementation significantly increased the hepatic bacteria clearance of SVC-injected *K*. *pneumoniae* in STZ-DM mice (Fig 3B). These results indicate that diabetes induces hepatic bacteria clearance impairment and FOS or dead L. salivarius feeding reverses it.

### Diabetes induced enteric bacteria translocation to liver and FOS or dead *L*. *salivarius* feeding reversed it

There was no bacterial translocation to liver in the control group. STZ-DM mice had a significant increase of 500 CFU/g bacteria translocation to liver as compared with the control group (Fig 3C). FOS or dead *L*. *salivarius* feeding decreased the enteric bacteria translocation to liver in STZ-DM mice (Fig 3C). These results indicate that diabetes induces enteric bacteria translocation to liver and FOS or dead *L*. *salivarius* feeding reverses it.

### Diabetes induced intraluminally injected *K*. *pneumoniae* pathogenic *K*. *pneumoniae* translocation to liver and FOS or dead *L*. *salivarius* feeding reversed it

To investigate effects of diabetes on intraluminally injected K. pne*umoniae* translocation to liver, *K*. *pneumoniae* counts of liver in STZ-DM mice were examined. STZ-DM mice demonstrated a significant 46.8-fold increase of translocation of intraluminally injected *K*. *pneumoniae* to liver as compared with the control group (Fig 3D). FOS or dead *L*. *salivarius* feeding significantly reduced the pathogenic *K*. *pneumoniae* translocation to liver in STZ-DM mice (Fig 3D). These results indicate that diabetes induces pathogenic *K*. *pneumoniae* translocation to liver and FOS or dead *L*. *salivarius* feeding reverses it.

### Diabetes induced iNOS protein expression of the intestinal mucosa and FOS or dead *L*. *salivarius* feeding decreased it

**Fig 4:**
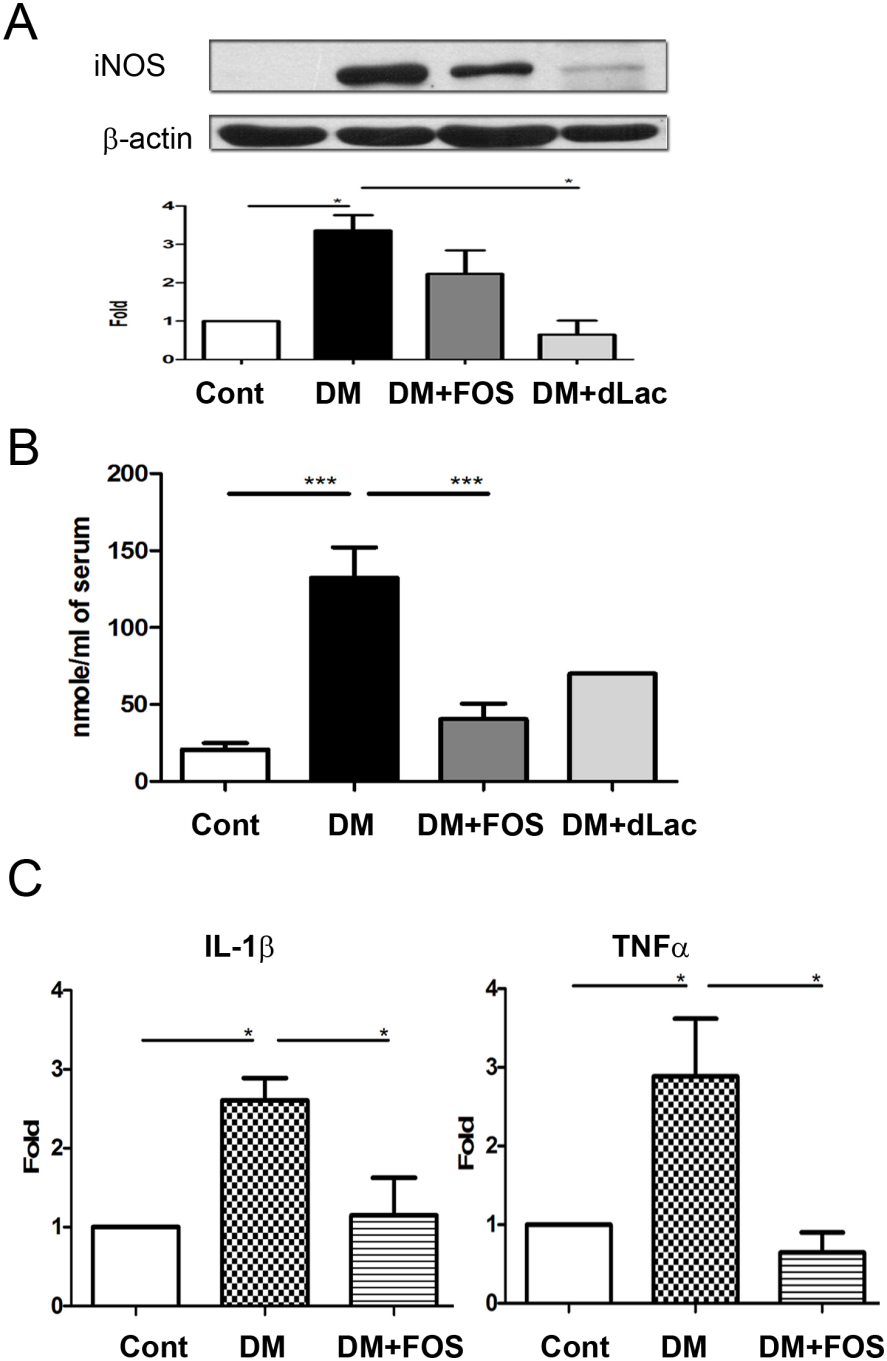
FOS or dead *L*. *salivarius* feeding reversed diabetes-induced iNOS expression of the intestinal mucosa, plasma NO levels in the portal vein, and IL-1β as well as TNF-α expression of the Kupffer cells in STZ-DM mice. (A) STZ-DM demonstrated a significant increase of iNOS protein expression of the intestinal mucosa as examined by Western blotting and FOS or dead *L*. *salivarius* feeding significantly decreased it. (B) STZ-DM demonstrated a significant increase of plasma NO levels of the portal vein as measured by Griess reagents and FOS or dead *L*. *salivarius* feeding significantly decreased them. (C) STZ-DM demonstrated a significant increase of IL-1β and TNF-α expression of Kupffer cells and FOS feeding significantly decreased them. STZ, streptozotocin; DM, diabetes mellitus; FOS, fructooligosaccharides; dLac, dead *L*. *salivarius*. *, *P*< 0.05; **, *P*< 0.01; ***, < 0.001. n = 6/group.

To examine the involvement of nitric oxide in diabetes-induced enteric bacterial translocation, protein expression of iNOS in the intestinal mucosa was assessed. STZ-DM demonstrated a significant increase of iNOS protein expression of the intestinal mucosa as compared with that in the control group (Fig 4A). FOS or dead *L*. *salivarius* feeding significantly decreased the iNOS expression of the intestinal mucosa in STZ-DM mice. Effects of dead *L*. *salivarius* on decreasing iNOS expression of the intestinal mucosa were much better than that of FOS (Fig 4A). These results indicate that diabetes induces iNOS protein expression of the intestinal mucosa and FOS or dead *L*. *salivarius* feeding decreases it.

### Diabetes induced plasma NO levels in portal vein and FOS feeding decreased it

STZ-DM mice demonstrated a significant 7-fold increase of plasma NO levels in the portal vein as compared with that in the control group. FOS feeding significantly decreased 70% plasma NO levels in the portal vein in STZ-DM mice. These results indicate that diabetes induces plasma NO levels in the portal vein and FOS feeding decreases it (Fig 4B).

### Diabetes induced IL-1β and TNF-α expression of Kupffer cells and FOS feeding reversed them

Increased cytokine production of Kupffer is closely related with hepatocyte injury and cholestasis [23]. To examine the changes of inflammatory cytokines of Kupffer cells in diabetes, IL-1β and TNF-α expression of Kupffer cells were examined. Diabetes induced a significant increase of IL-1β and TNF-α expression of Kupffer cells in STZ-DM mice as compared with the control group (Fig 4C). FOS feeding significantly decreased IL-1β and TNF-α expression of Kupffer cells in STZ-DM mice. These results indicate that diabetes-induced IL-1β and TNF-α expression of Kupffer cells and FOS feeding reverses it.

### L-NAME but not D-NAME decreased iNOS expression of the intestinal mucosa in STZ-DM mice

**Fig 5:**
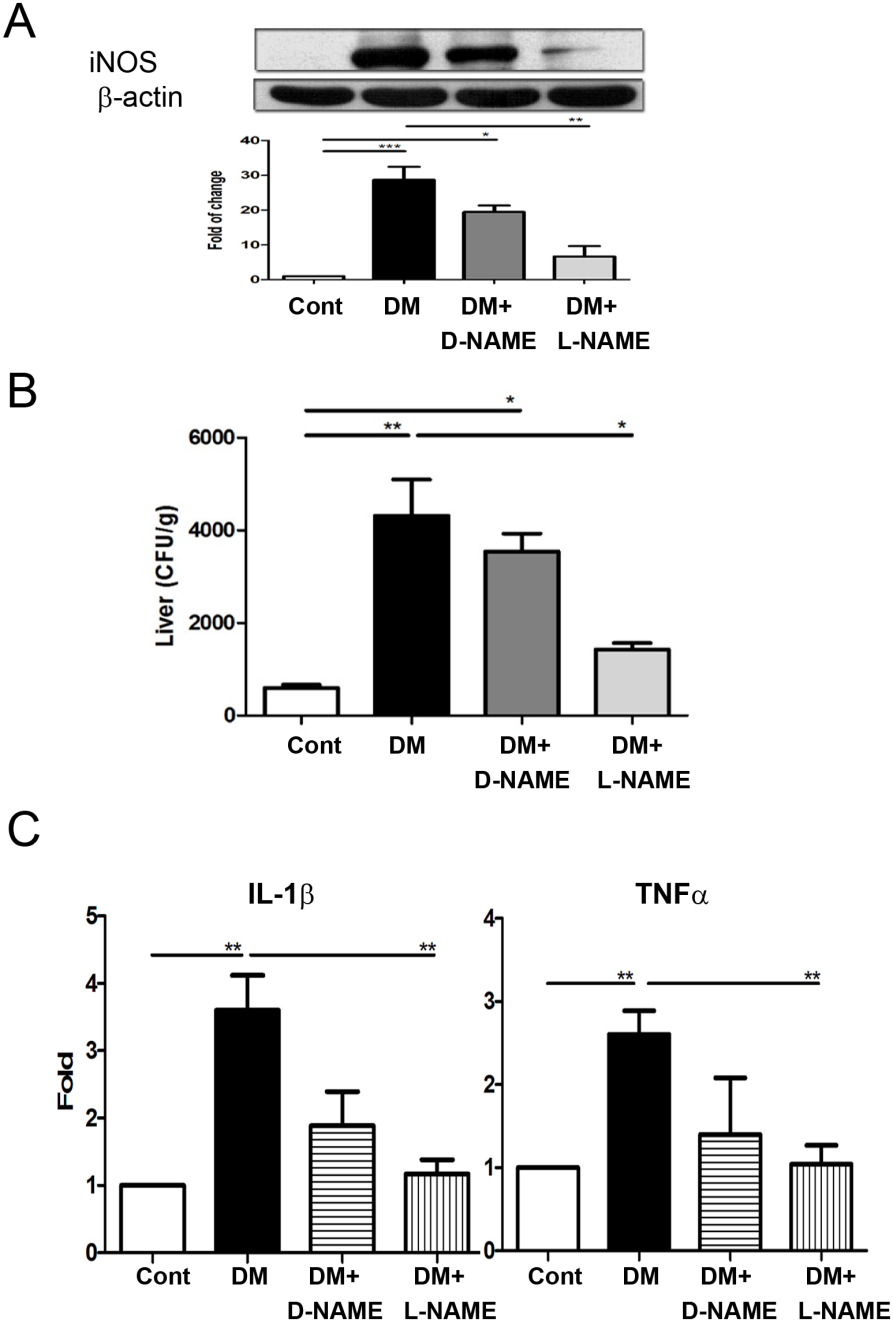
L-NAME but not D-NAME decreased iNOS expression of the intestinal mucosa, hepatic bacteria clearance, and IL-1β as well as TNF-α expression of the Kupffer cells in STZ-DM mice. (A) STZ-DM mice demonstrated a significant increase of iNOS protein expression of the intestinal mucosa as compared with that in the WT group. L-NAME but not D-NAME supplementation decreased iNOS protein expression of the intestinal mucosa in STZ-DM mice. (B) L-NAME but not D-NAME feeding decreased the bacterial loads of liver in STZ-DM mice. STZ-DM mice were fed with an L-NAME (0.5 mg/ml) or D-NAME (0.5 mg/ml, as a control drug) in drinking water for one week. *K*. *pneumoniae* (1 × 10^3^ CFU, K2 serotype) were injected into the branch of superior mesenteric vein (SVC). The liver was collected, weighed and homogenized at 4 hr after the injection for bacterial culture. (C) STZ-DM mice demonstrated a significant increase of IL-1β and TNF-α expression of the Kupffer cells. L-NAME but not D-NAME supplementation markedly decreased diabetes-induced IL-1β and TNF-α mRNA expression of Kupffer cells in STZ-DM mice. STZ, streptozotocin; L-NAME, L-NG-Nitroarginine Methyl Ester; DM, diabetes mellitus; FOS, fructooligosaccharides; dLac, dead *L*. *salivarius*. *, *P*< 0.05; **, *P*< 0.01; ***, < 0.001. n = 6/group.

To examine the link between NO production and hepatic defense impairment in diabetes, animals were fed with NOS inhibitor, L-NAME, to block the iNOS expression of the intestinal mucosa. STZ-DM mice demonstrated a significant increase of iNOS protein expression of the intestinal mucosa as compared with the WT group. iNOS protein expression of the intestinal mucosa was significantly decreased by L-NAME but not by D-NAME supplementation as measured by Western blotting (Fig 5A). These results indicate that L-NAME but not D-NAME supplementation decreases diabetes-induced iNOS protein expression of the intestinal mucosa.

### L-NAME supplementation reversed diabetes-induced hepatic bacteria clearance impairment in STZ-DM mice

Diabetes induced a significant decrease of hepatic bacteria clearance in STZ-DM mice. The bacterial loads of liver in the STZ-DM + L-NAME group showed a significant 68% decrease as compared with that in the STZ-DM group (Fig 5B). Orally feeding of D-NAME showed no significant effect on diabetes-induced hepatic bacteria clearance in STZ-DM mice (Fig 5B). These results indicate that diabetes reduces hepatic bacteria clearance against *K*. *pneumoniae* translocation and iNOS inhibition supplementation reverses it.

### L-NAME supplementation decreased IL-1β and TNF-α expression of Kupffer cells in STZ-DM mice

Diabetes significantly induced IL-1β and TNF-α expression of the Kupffer cells in STZ-DM mice (Fig 5C). L-NAME but not D-NAME supplementation markedly decreased diabetes-induced IL-1β and TNF-α mRNA expression of Kupffer cells in STZ-DM mice (Fig 5C). These results indicate that diabetes induces IL-1β as well as TNF-α expression of Kupffer cells and iNOS inhibition decreases it.

### FOS feeding reversed diabetes-induced hepatic bacterial clearance impairment in Ins2^Akita^ mice

**Fig 6:**
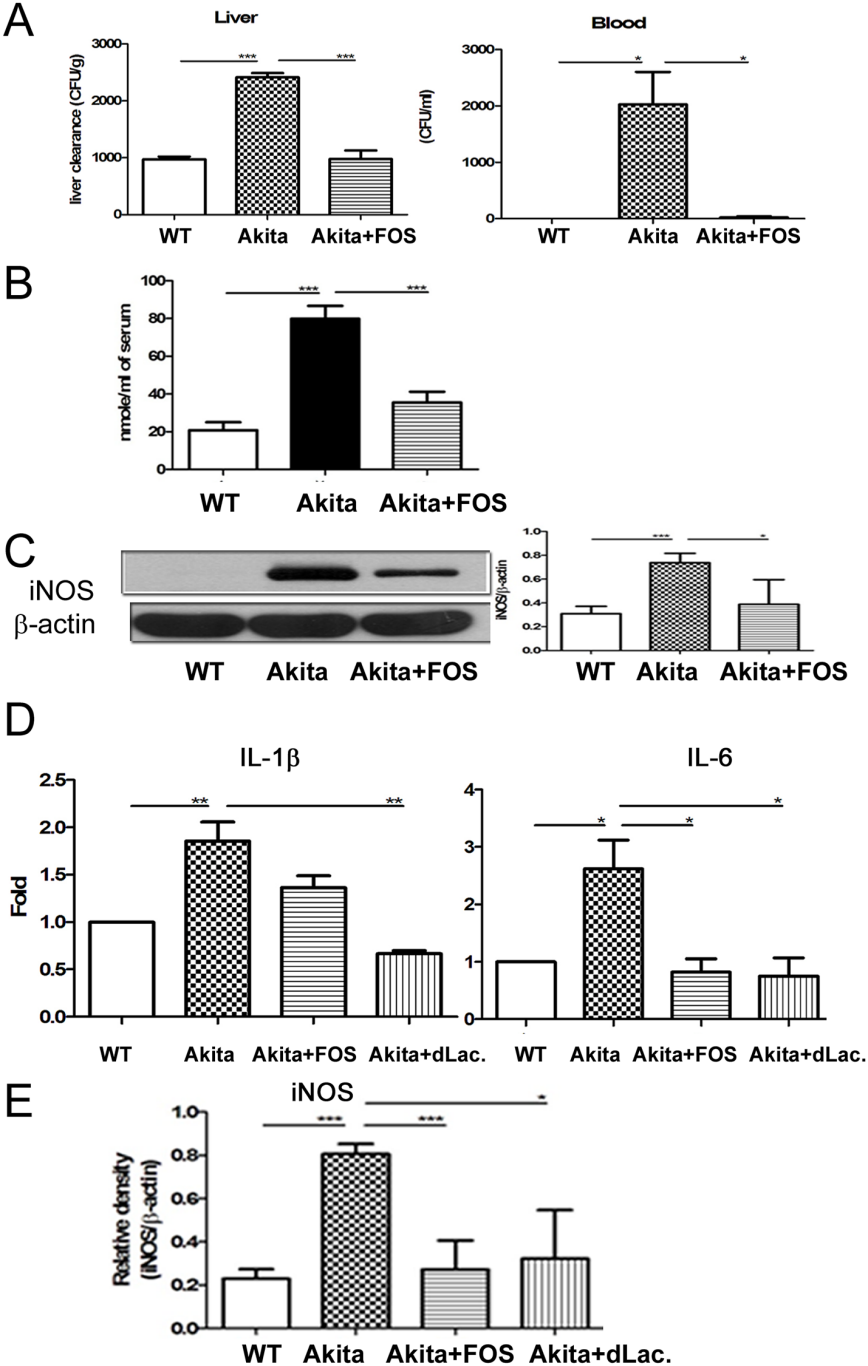
FOS feeding reversed diabetes-induced hepatic bacterial clearance impairment, IL-1β expression of the Kupffer, and iNOS expression of the intestinal mucosa in Ins2^Akita^ mice. (A) Ins2^Akita^ mice demonstrated a significant increase of *K*. *pneumoniae* translocation to liver and blood as compared with that in WT group. FOS supplementation significantly increased hepatic bacteria clearance and decreased K. pneumonia translocation to liver as well as blood in Ins2^Akita^ mice. *K*. *pneumoniae* (1 × 10^3^ CFU, K2 serotype) were injected into the branch of superior mesenteric vein (SVC). The liver was collected, weighed and homogenized in 1 ml of sterile saline at 4 hr after the injection. 100 μl of blood were taken from heart. Blood or aliquots of the homogenates were plated onto tryptic soy broth (TSB) agar plates (DIFCO). (B) Diabetes induced plasma NO levels in the portal vein as measured by Griess reagents and FOS feeding decreased them. (C) Diabetes significantly induced iNOS protein expression of the intestinal mucosa in Ins2^Akita^ mice and FOS feeding decreased it. (D) Diabetes induces a significant increase of IL-1β as well as IL6 expression of Kupffer cells in Ins2^Akita^ mice. FOS or dead *L*. *salivarius* supplementation markedly decreased it. (E) Diabetes induced iNOS mRNA expression of the intestinal mucosa in Ins2^Akita^ mice. FOS or dead *L*. *salivarius* supplementation decreased it. STZ, streptozotocin; DM, diabetes mellitus; FOS, fructooligosaccharides; dLac, dead *L*. *salivarius*. *, *P*< 0.05; **, *P*< 0.01; ***, < 0.001. n = 6/group.

Ins2^Akita^ mice demonstrated a significant 2.5-fold and 2000 CFU/g increase of *K*. *pneumoniae* translocation to liver and blood respectively as compared with those in the WT group (Fig 6A). FOS supplementation significantly increased hepatic bacteria clearance and decreased K. pneumonia translocation to liver as well as blood in Ins2^Akita^ mice (Fig 6A). These results corroborate that diabetes induces hepatic bacteria clearance impairment and FOS supplementation reverses it.

### FOS feeding decreased plasma NO levels and intestinal iNOS expression in Ins2^Akita^ mice

Diabetes induced a significant 4-fold increase of plasma NO levels in the portal vein in Ins2^Akita^ mice as compared with the WT mice. FOS treatment induced a significant 57% decrease of plasma NO levels in the portal vein in Ins2^Akita^ mice (Fig 6B). Moreover, diabetes significantly induced iNOS protein expression of the intestinal mucosa in Ins2^Akita^ mice and FOS treatment decreases it (Fig 6C).

### FOS or dead *L*. *salivarius* feeding reversed diabetes-induced cytokine expression of Kupffer cells in Ins2^Akita^ mice

To further corroborate the stimulatory effects of diabetes on Kupffer cells, cytokine expression of Kupffer cells in Ins2^Akita^ mice was examined. Diabetes induces a significant increase of IL-1β and IL-6 expression of Kupffer cells in Ins2^Akita^ mice. FOS supplementation decreased IL-1β expression and dead *L*. *salivarius* supplementation decreased IL-1β as well as IL-6 expression of Kupffer cells in Ins2^Akita^ mice (Fig 6D).

### FOS or dead *L*. *salivarius* feeding reversed diabetes-induced intestinal iNOS mRNA expression in Ins2^Akita^ mice

Diabetes significantly induced iNOS mRNA expression of the intestinal mucosa in Ins2^Akita^ mice. FOS or dead *L*. *salivarius* supplementation decreased iNOS mRNA expression in Ins2^Akita^ mice (Fig 6E).

### Dead bacteria feeding induced TLR4 expression of intestinal mucosa in germ-free mice

**Fig 7:**
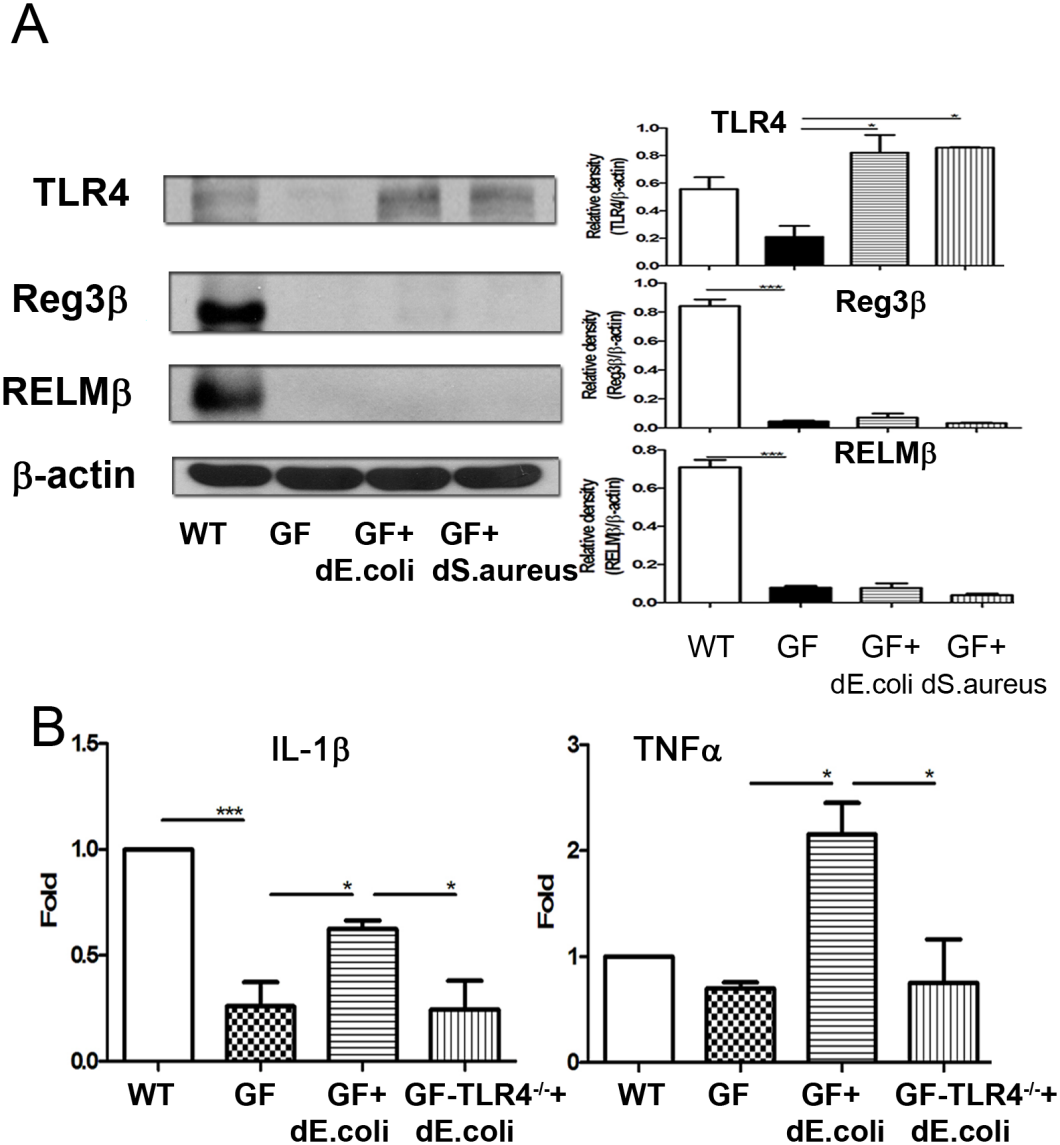
Dead *E*. *coli* feeding induced TLR4 expression of the intestinal mucosa and IL-1β as well as TNF-α expression of Kupffer cells. (A) Dead *E*. *coli* or *S*. *aureus* feeding for one week significantly induced TLR4 protein expression of the intestinal mucosa in germ-free mice. Germ free mice demonstrated a significant decrease of iNOS protein expression of the intestinal mucosa as compared with those in SPF mice. (B) Dead *E*. *coli* feeding for one week significantly induced IL-1β and TNF-α expression of Kupffer cells in germ-free mice but not in germ-free TLR4^-/-^ mice. dE.coli, dead *E*.*coli*; GF, germ-free mice; GF-TLR4^-/-^; germ-free TLR4^-/-^ mice. *, *P*< 0.05; **, *P*< 0.01; ***, < 0.001. n = 5/group.

To examine whether bacteria could induce TLR4, Reg3β, and RELMβ expression of the intestinal mucosa, germ-free mice were orally fed with dead *E*.*coli* or *S*. *aureus*. A broad range of antimicrobial proteins such as enteric Reg3β and RELMβ could be synthesized by Paneth cells to limit bacterial penetration [24]. Germ-free mice demonstrated a significant decrease of TLR4, Reg3β, and RELMβ protein expression of the intestinal mucosa as compared with SPF mice (S1 Fig). Dead *E*. *coli* feeding significantly induced TLR4 protein expression of the intestinal mucosa in germ-free mice. Dead *E*. *coli* or *S*. *aureus* feeding for one week significantly induced TLR4 protein expression of the intestinal mucosa in germ-free mice (Fig 7A). These results suggest that bacteria are important in inducing TLR4, Reg3β, and RELMβ expression of the intestinal mucosa. Dead bacteria feeding induces TLR4 protein expression of the intestinal mucosa.

### Dead *E*. *coli* feeding induced IL-1β and TNF-α expression of Kupffer cells in germ-free mice but not in germ-free TLR4^-/-^ mice

To examine whether microbiota could induces IL-1β expression of Kupffer cells, cytokine expression of Kupffer cells in germ-free and germ-free TLR4^-/-^ mice was examined. Germ-free mice demonstrated a significant decrease of IL-1β expression of Kupffer cells as compared with that in the SPF mice (Fig 7B). Dead *E*. *coli* feeding for one week significantly induced IL-1β and TNF-α expression of Kupffer cells in germ-free mice but not in germ-free TLR4^-/-^ mice (Fig 7B). These results indicate that microbiota are important in inducing IL-1β expression of Kupffer cells and intestinal *E*. *coli* could induce hepatic Kupffer cell activation through TLR4.

## Discussion

There is limited knowledge regarding the pathological mechanisms of how *K*. *pneumoniae* infects the liver in diabetic patients. Originally, increased plasma ALT levels and decreased hepatic bacteria clearance were observed in STZ-DM mice, both observations suggested that liver damage, inflammation and evolving sepsis have happened in diabetic mice. Emerging evidences have suggested that there is a strong interaction between the gut microbiota and the liver function. Bacterial translocation was more frequently found in cirrhotic patients with small intestinal bacterial overgrowth as compared to patients without overgrowth [25]. When bacterial overgrowth is induced in the small intestine experimentally, it results in hepatic injury mediated by translocated bacterial products [26]. Consistent with these studies, increased intestinal BT especially pathogenic *K*. *pneumoniae* (K2) was observed in STZ-DM mice. The results of increased BT were not only confirmed by pathogenic *K*. *pneumoniae* (K2) feeding but also by pathogenic *K*. *pneumoniae* (K2) injection to the lumen. It has known BT may occur as a consequence of altered bacterial overgrowth and host defense mechanisms. Bacterial overgrowth frequently occurs in cirrhosis and it appears to be related to the degree of hepatic dysfunction [27]. Then, as described, bacterial overgrowth of total aerobic bacteria in intestinal lumen occurred in the diabetic mice. Through specifically qualitative and quantitative analyzing, advanced findings of enteric dysbiosis were also observed. There was an overall increase of some pathogenic bacteria including aerobic bacteria *Enterobacteriaceae*, *Enterococcus*, *K*. *pneumoniae*, *E*. *coli*, *Staphylococcus* and anaerobic bacteria *Bacteroids* in diabetic mice. Interestingly, diabetic mice showed significantly less *Lactobacillus* one month after STZ treatment. All above results demonstrated that the intestinal homeostasis have broken down in diabetic mice. The ethanol-induced enteric dysbiosis and liver injury could be improved by the beneficial effects of probiotic *Lactobacillus* strains to displace Gram-negative bacteria and subsequently reduce the systemic endotoxin levels in rats [28]. Patients treated with probiotics had a restoration of the gut flora with an increased number of both *Bifidobacteria* and *Lactobacilli*, compared to controls [4]. Therefore, in this study, mice were respectively fed with prebiotic to stimulate *Lactobacillus* strains proliferation and dead *L*. *salivarius* to directly activate mucosal defense in a manner similar to endogenous probiotics against pathogens. Our results showed both treatments significantly decreased luminal and mucosal bacteria overgrowth, and further alleviated diabetes-induced enteric dysbiosis that resulted in decreased pathogenic bacteria including aerobic bacteria *Enterococcus*, *K*. *pneumoniae*, *E*. *coli*, *Staphylococcus* as well as anaerobic bacteria *Bacteroids* and increased *Lactobacillus* in diabetic mice. The significant decreased translocation of intestinal bacteria and injected *K*. *pneumoniae* (K2) to liver was also occurred in diabetic mice fed with these two treatments for one week. Further, in the advanced findings of cytokine expression of the Kupffer cells, the mRNA expression of IL-1β and TNF-α expression was significantly reduced in diabetic mice by feeding with the both treatments. Following the alleviated intestinal dysbiosis, the liver injury in diabetic mice was significantly restored according to the decreased plasma ALT levels and increased hepatic bacteria clearance. Altogether, our findings suggest that diabetes induces *K*. *pneumoniae* liver abscess through the induction of intestinal dysbiosis, liver injury, *K*. *pneumoniae* translocation to liver, and decreased hepatic bacteria clearance. Prebiotic as well as dead *L*. *salivarius* supplementation decrease diabetes-induced intestinal dysbiosis, liver injury, and *K*. *pneumoniae* translocation to liver. Our data suggests that prebiotic as well as dead *L*. *salivarius* supplementation may be useful to prevent *K*. *pneumoniae* liver abscess as well as liver injury in diabetes patients.

The mechanisms of the increase of liver abscess in diabetes included the increase of bacterial translocation from intestine to liver and the increase of bacterial stasis in liver. First, we examined the bacterial translocation from intestine to liver by examining bacterial culture of liver after injecting *K*. *pneumoniae* into the intestinal lumen. Next, we examined the bacterial stasis in liver by examining bacterial culture of liver after injecting *K*. *pneumoniae* into the superior mesenteric vein. To further examine the different roles of liver and gut in diabetes-induced *K*. *pneumoniae* liver abscess, we injected *K*. *pneumoniae* to mesenteric vein. Interestingly, the *K*. *pneumonia* loads in the liver after *K*. *pneumoniae* injection in mesenteric vein were significantly increased in STZ-DM and Ins2^Akita^ mice. Moreover, IL-1β expression of the Kupffer cells was significantly increased in STZ-DM and Ins2^Akita^ mice. These results indicate that diabetes induces *K*. *pneumonia* liver abscess through at least two mechanisms. First, diabetes induces intestinal dysbiosis and *K*. *pneumoniae* translocation. Second, diabetes induces NO levels in the portal vein and subsequent inflammation of Kupffer cells. Increased cytokine production of Kupffer is closely related with hepatocyte injury and cholestasis [23]. Altogether, diabetes induces intestinal dysbiosis, *K*. *pneumoniae* translocation to liver, activation of Kupffer cells, and subsequent *K*. *pneumoniae* liver abscess.

Oxygen free radicals are known to play an important role in the gut epithelial damage, which may alter the gut barrier function, facilitate BT and release of endotoxin [29]. An overexpression of NO following the activation of iNOS, eNOS and nNOS contribute to the pathogenic role in many liver diseases resulting in portal hypertension [4]. These enzymes are mainly activated by LPS, pro-inflammatory cytokines and endotoxin as demonstrated in several studies [30]. The Kupffer cells, recruited macrophages, and inflammatory cells results in the production of cytokines and chemokines that lead to prolonged inflammation and hepatocyte damage [31]. Both STZ-DM and Ins2^Akita^ mice demonstrated a significant increase of intestinal iNOS protein expression compared to control mice. And then, increased plasma NO levels and IL-1β as well as TNF-α expression of Kupffer cells was subsequently detected in the diabetic mice. Interestingly, all of above effects causing by the nitrosative stress were reversed after treating with prebiotic or dead *L*. *salivarius* feeding including plasma ALT level and hepatic bacteria clearance in diabetic mice. According to the attenuated effects of intestinal NO by prebiotic and dead *L*. *salivarius* feeding in diabetic mice, it may be hypothesized that inhibition of iNOS expression could prevent intestinal barrier dysfunction and hepatic inflammation in diabetic mice. Our results demonstrated that plasma NO levels in the portal vein were significantly decreased when intestinal iNOS expression was inhibited by L-NAME but not D-NAME in diabetic mice. Following the attenuated gut NO production, IL-1β and TNF-α expression of the Kupffer cells and K. pneumonia loads of the liver were decreased. These results suggest that NO production from intestinal tract plays an important role in inducing IL-1β and TNF-α expression of the Kupffer cells and the following *K*. *pneumoniae* stasis in the liver. Diabetes induces intestinal iNOS expression, plasma NO levels in the portal vein, IL-1β and TNF-α expression of Kupffer cells, and K. pneumonia loads in the liver. iNOS inhibition by L-NAME decreases plasma NO levels in the portal vein, IL-1β and TNF-α expression of Kupffer cells, and K. pneumonia loads in the liver. Altogether, our results suggest that iNOS inhibitor could be useful in decreasing diabetes-induced *K*. *pneumoniae* liver abscess.

TLRs represent a link between intestinal flora changes, endotoxemia, and liver damage [5]. Our data demonstrated a significant decrease of TLR4 expression of the intestinal mucosa and IL-1β expression of Kupffer cells in GF mice. Dead *E*.*coli* or *S*. *aureus* feeding induced TLR4 expression of the intestinal mcosa. These results suggest that bacteria are important in inducing TLR4 expression of the intestinal mucosa. Moreover, dead *E*.*coli* feeding induces IL-1β and TNF-α expression of Kupffer cells in germ-free mice but not in germ-free TLR4^-/-^ mice. Altogether, our results suggest that *E*.*coli* could induce IL-1β and TNF-α expression of Kupffer cells through intestinal toll-like receptor 4.

**Fig 8:**
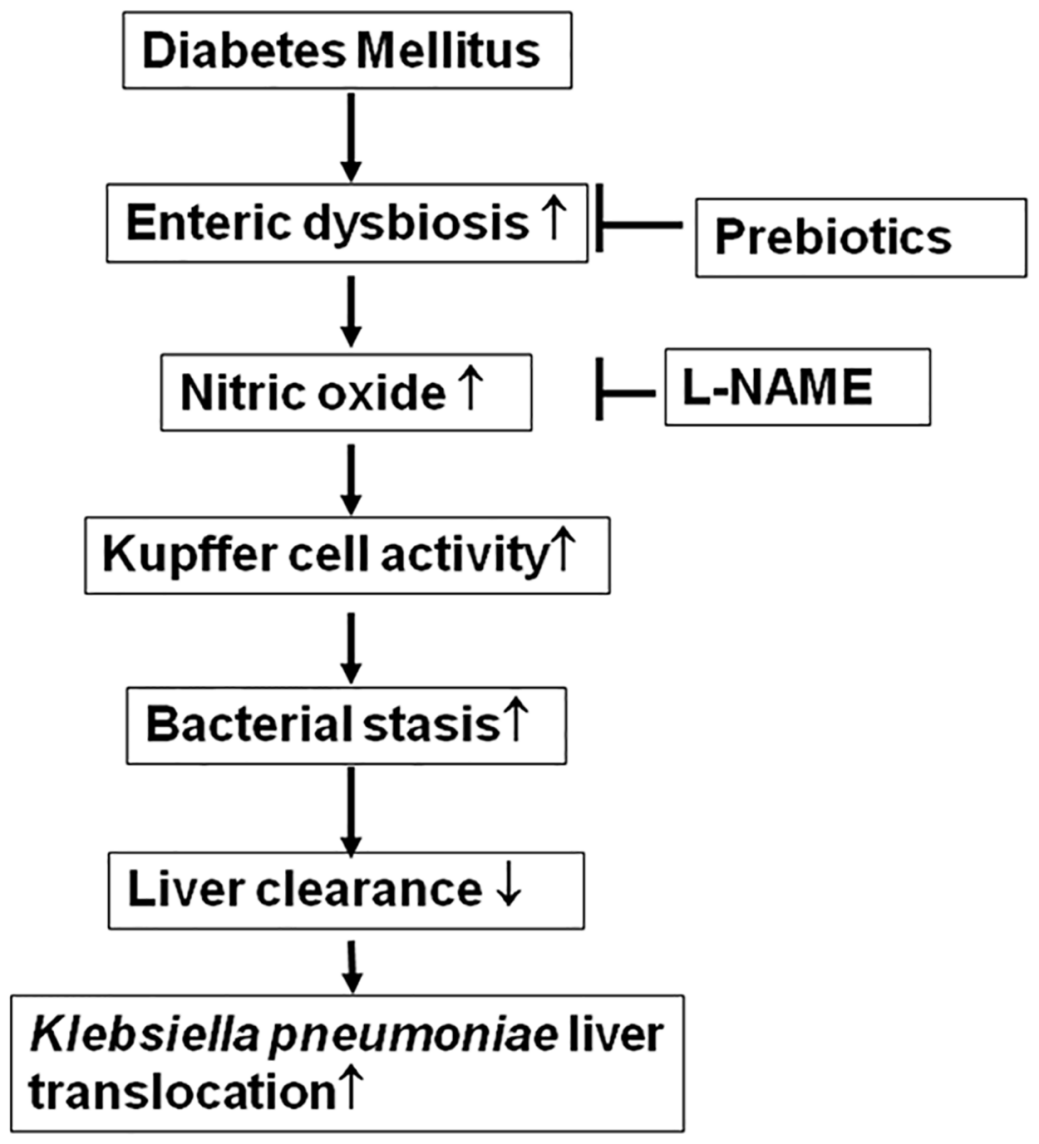
The regulatory mechanism of enteric dysbiosis on Kupffer cell activation and *Klebsiella pneumonia* liver translocation in diabetes. L-NAME, L-NG-Nitroarginine Methyl Ester.

In conclusion, diabetes induces intestinal bacterial overgrowth, enteric dysbiosis, bacterial translocation (especially the translocation of pathogen *K*. *pneumoniae*), intestinal iNOS protein expression, NO levels in the portal vein, activation of Kupffer cells, and *K*. *pneumoniae* bacterial loads in the liver. The effect of diabetes on *K*. *pneumoniae* translocation to liver could be attenuated by correcting enteric dysbiosis with prebiotic (FOS) and probiotic (dead *L*. *salivarius*) treatment or by inhibition of gut iNOS protein expression with L-NAME (Fig 8). Feeding of dead bacteria induces Kupffer cells activation through toll-like receptor 4.

## Supporting information

S1 Fig Dead *E*. *coli* or *S*. *aureus* feeding for one week did not induce Reg3β or RELMβ protein expression of the intestinal mucosa in germ-free mice. Germ free mice demonstrated a significant decrease of Reg3β and RELMβ protein expression of the intestinal mucosa as compared with those in SPF mice. ***, *P*< 0.001. n = 5/group.
(TIF)

## References

[1] Lin YC, Lu MC, Tang HL, Liu HC, Chen CH, Liu KS, et al Assessment of hypermucoviscosity as a virulence factor for experimental Klebsiella pneumoniae infections: comparative virulence analysis with hypermucoviscosity-negative strain. BMC Microbiol. 2011;11:50 10.1186/1471-2180-11-50 21385400 PMC3060850

[2] Wang JH, Liu YC, Lee SS, Yen MY, Chen YS, Wann SR, et al Primary liver abscess due to Klebsiella pneumoniae in Taiwan. Clin Infect Dis. 1998;26(6):1434–8. 9636876 10.1086/516369

[3] Fung CP, Chang FY, Lin JC, Ho DM, Chen CT, Chen JH, et al Immune response and pathophysiological features of Klebsiella pneumoniae liver abscesses in an animal model. Lab Invest. 2011;91(7):1029–39. 10.1038/labinvest.2011.52 21464821

[4] Cesaro C, Tiso A, Del Prete A, Cariello R, Tuccillo C, Cotticelli G, et al Gut microbiota and probiotics in chronic liver diseases. Dig Liver Dis. 2011;43(6):431–8. 10.1016/j.dld.2010.10.015 21163715

[5] Szabo G, Dolganiuc A, Mandrekar P. Pattern recognition receptors: a contemporary view on liver diseases. Hepatology. 2006;44(2):287–98. 10.1002/hep.21308 16871558

[6] Seki E, Schnabl B. Role of innate immunity and the microbiota in liver fibrosis: crosstalk between the liver and gut. J Physiol. 2012;590(Pt 3):447–58. 22124143 10.1113/jphysiol.2011.219691 PMC3379693

[7] Chung PH, Wu YY, Chen PH, Fung CP, Hsu CM, Chen LW. Lactobacillus salivarius reverse diabetes-induced intestinal defense impairment in mice through non-defensin protein. J Nutr Biochem. 2016;35:48–57. 10.1016/j.jnutbio.2016.05.013 27376728

[8] Henao-Mejia J, Elinav E, Jin C, Hao L, Mehal WZ, Strowig T, et al Inflammasome-mediated dysbiosis regulates progression of NAFLD and obesity. Nature. 2012;482(7384):179–85. 10.1038/nature10809 22297845 PMC3276682

[9] Miura K, Kodama Y, Inokuchi S, Schnabl B, Aoyama T, Ohnishi H, et al Toll-like receptor 9 promotes steatohepatitis by induction of interleukin-1beta in mice. Gastroenterology. 2010;139(1):323–34 e7. 10.1053/j.gastro.2010.03.052 20347818 PMC4631262

[10] Moro G, Minoli I, Mosca M, Fanaro S, Jelinek J, Stahl B, et al Dosage-related bifidogenic effects of galacto- and fructooligosaccharides in formula-fed term infants. J Pediatr Gastroenterol Nutr. 2002;34(3):291–5. 11964956 10.1097/00005176-200203000-00014

[11] Alican I, Kubes P. A critical role for nitric oxide in intestinal barrier function and dysfunction. Am J Physiol. 1996;270(2 Pt 1):G225–37. 8779963 10.1152/ajpgi.1996.270.2.G225

[12] Unno N, Wang H, Menconi MJ, Tytgat SH, Larkin V, Smith M, et al Inhibition of inducible nitric oxide synthase ameliorates endotoxin- induced gut mucosal barrier dysfunction in rats [see comments]. Gastroenterology. 1997;113(4):1246–57. 9322519 10.1053/gast.1997.v113.pm9322519

[13] Tang Y, Forsyth CB, Farhadi A, Rangan J, Jakate S, Shaikh M, et al Nitric oxide-mediated intestinal injury is required for alcohol-induced gut leakiness and liver damage. Alcohol Clin Exp Res. 2009;33(7):1220–30. 10.1111/j.1530-0277.2009.00946.x 19389191 PMC2950098

[14] Yan AW, Schnabl B. Bacterial translocation and changes in the intestinal microbiome associated with alcoholic liver disease. World J Hepatol. 2012;4(4):110–8. 10.4254/wjh.v4.i4.110 22567183 PMC3345535

[15] Barber AJ, Antonetti DA, Kern TS, Reiter CE, Soans RS, Krady JK, et al The Ins2Akita mouse as a model of early retinal complications in diabetes. Invest Ophthalmol Vis Sci. 2005;46(6):2210–8. 10.1167/iovs.04-1340 15914643

[16] Sierra S, Lara-Villoslada F, Sempere L, Olivares M, Boza J, Xaus J. Intestinal and immunological effects of daily oral administration of Lactobacillus salivarius CECT5713 to healthy adults. Anaerobe. 2010;16(3):195–200. 10.1016/j.anaerobe.2010.02.001 20159049

[17] Nemzek JA, Xiao HY, Minard AE, Bolgos GL, Remick DG. Humane endpoints in shock research. Shock. 2004;21(1):17–25. 10.1097/01.shk.0000101667.49265.fd 14676679

[18] Momozawa Y, Deffontaine V, Louis E, Medrano JF. Characterization of bacteria in biopsies of colon and stools by high throughput sequencing of the V2 region of bacterial 16S rRNA gene in human. PLoS One. 2011;6(2):e16952 10.1371/journal.pone.0016952 21347324 PMC3037395

[19] Hunninghake GW, Doerschug KC, Nymon AB, Schmidt GA, Meyerholz DK, Ashare A. Insulin-like growth factor-1 levels contribute to the development of bacterial translocation in sepsis. Am J Respir Crit Care Med. 2010;182(4):517–25. 10.1164/rccm.200911-1757OC 20413631 PMC2937242

[20] Ashare A, Monick MM, Powers LS, Yarovinsky T, Hunninghake GW. Severe bacteremia results in a loss of hepatic bacterial clearance. Am J Respir Crit Care Med. 2006;173(6):644–52. 10.1164/rccm.200509-1470OC 16399991 PMC2662948

[21] Sakai N, Van Sweringen HL, Schuster R, Blanchard J, Burns JM, Tevar AD, et al Receptor activator of nuclear factor-kappaB ligand (RANKL) protects against hepatic ischemia/reperfusion injury in mice. Hepatology. 2012;55(3):888–97. 10.1002/hep.24756 22031462 PMC3276725

[22] Matsuguchi T, Musikacharoen T, Ogawa T, Yoshikai Y. Gene expressions of Toll-like receptor 2, but not Toll-like receptor 4, is induced by LPS and inflammatory cytokines in mouse macrophages. J Immunol. 2000;165(10):5767–72. 11067935 10.4049/jimmunol.165.10.5767

[23] El Kasmi KC, Anderson AL, Devereaux MW, Fillon SA, Harris JK, Lovell MA, et al Toll-like receptor 4-dependent Kupffer cell activation and liver injury in a novel mouse model of parenteral nutrition and intestinal injury. Hepatology. 2012;55(5):1518–28. 10.1002/hep.25500 22120983 PMC4986925

[24] Meyer-Hoffert U, Hornef MW, Henriques-Normark B, Axelsson LG, Midtvedt T, Putsep K, et al Secreted enteric antimicrobial activity localises to the mucus surface layer. Gut. 2008;57(6):764–71. 10.1136/gut.2007.141481 18250125

[25] Jun DW, Kim KT, Lee OY, Chae JD, Son BK, Kim SH, et al Association between small intestinal bacterial overgrowth and peripheral bacterial DNA in cirrhotic patients. Dig Dis Sci. 2010;55(5):1465–71. 10.1007/s10620-009-0870-9 19517230

[26] Lichtman SN, Sartor RB, Keku J, Schwab JH. Hepatic inflammation in rats with experimental small intestinal bacterial overgrowth. Gastroenterology. 1990;98(2):414–23. 2295397 10.1016/0016-5085(90)90833-m

[27] Bauer TM, Schwacha H, Steinbruckner B, Brinkmann FE, Ditzen AK, Aponte JJ, et al Small intestinal bacterial overgrowth in human cirrhosis is associated with systemic endotoxemia. Am J Gastroenterol. 2002;97(9):2364–70. 10.1111/j.1572-0241.2002.05791.x 12358257

[28] Mutlu E, Keshavarzian A, Engen P, Forsyth CB, Sikaroodi M, Gillevet P. Intestinal dysbiosis: a possible mechanism of alcohol-induced endotoxemia and alcoholic steatohepatitis in rats. Alcohol Clin Exp Res. 2009;33(10):1836–46. 10.1111/j.1530-0277.2009.01022.x 19645728 PMC3684271

[29] Parks DA, Bulkley GB, Granger DN. Role of oxygen-derived free radicals in digestive tract diseases. Surgery. 1983;94(3):415–22. 6351311

[30] Guarner C, Soriano G, Tomas A, Bulbena O, Novella MT, Balanzo J, et al Increased serum nitrite and nitrate levels in patients with cirrhosis: relationship to endotoxemia. Hepatology. 1993;18(5):1139–43. 8225220

[31] Szabo G, Mandrekar P, Dolganiuc A. Innate immune response and hepatic inflammation. Semin Liver Dis. 2007;27(4):339–50. 10.1055/s-2007-991511 17979071

