# Supplementary figures and images for: Inhibition of nitric oxide production reverses diabetes-induced Kupffer cell activation and Klebsiella pneumonia liver translocation
Source: PLoS One. 2017 May 11;12(5):e0177269. doi: 10.1371/journal.pone.0177269 (PMC5426676; doi:10.1371/journal.pone.0177269)

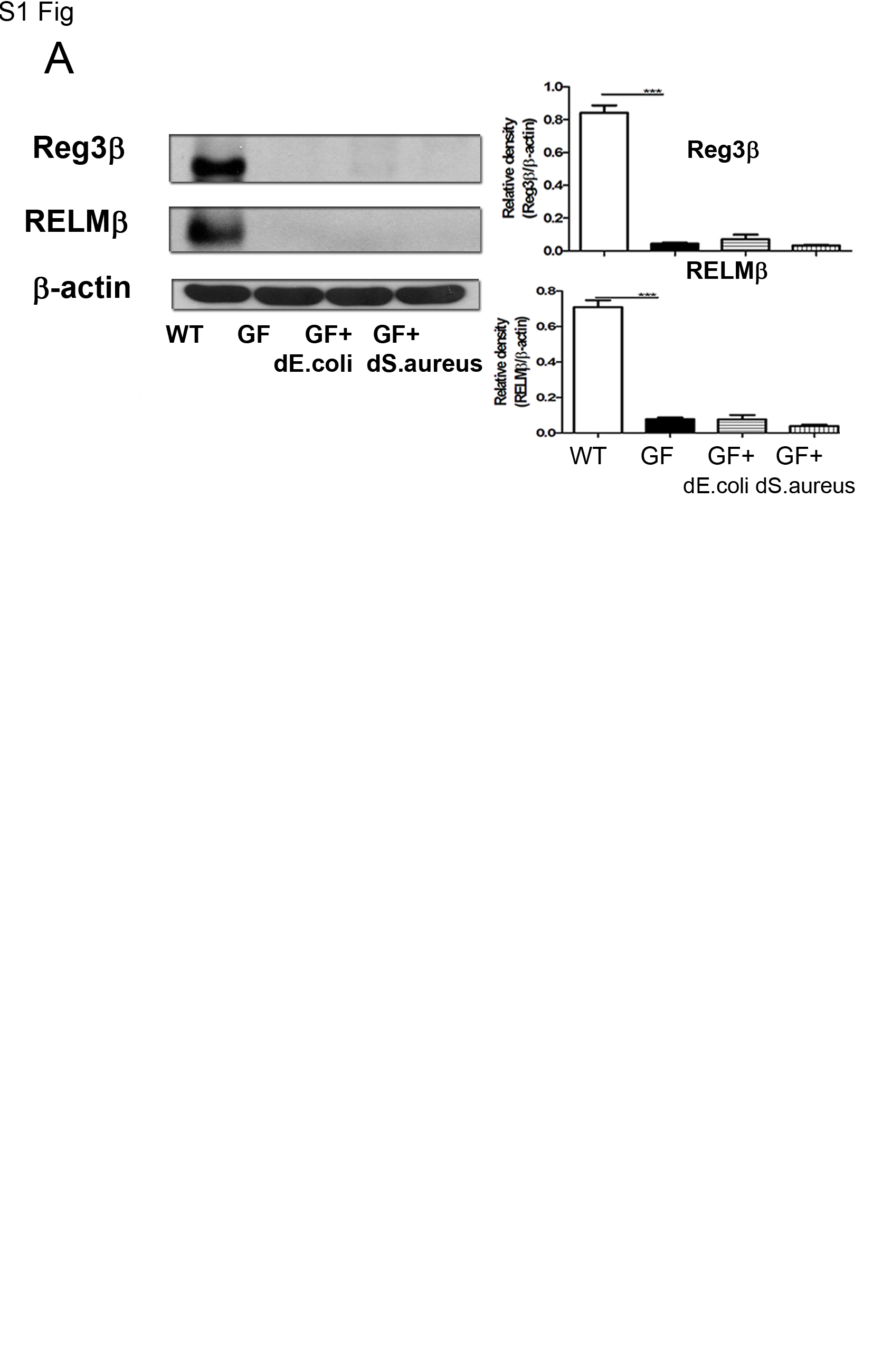

Supplement: S1 Fig — Germ free mice demonstrated a significant decrease of Reg3β and RELMβ protein expression of the intestinal mucosa as compared with those in SPF mice. ***, P< 0.001. n = 5/group. (TIF) [file pone.0177269.s001.tif]
